# SEMA: Antigen B-cell conformational epitope prediction using deep transfer learning

**DOI:** 10.3389/fimmu.2022.960985

**Published:** 2022-09-15

**Authors:** Tatiana I. Shashkova, Dmitriy Umerenkov, Mikhail Salnikov, Pavel V. Strashnov, Alina V. Konstantinova, Ivan Lebed, Dmitriy N. Shcherbinin, Marina N. Asatryan, Olga L. Kardymon, Nikita V. Ivanisenko

**Affiliations:** ^1^ Artificial Intelligence Research Institute, Moscow, Russia; ^2^ Sber AI Lab, Moscow, Russia; ^3^ AI Center Block Services, Sber, Moscow, Russia; ^4^ Federal Research Centre of Epidemiology and Microbiology named after Honorary Academician N. F. Gamaleya, Ministry of Health, Moscow, Russia; ^5^ Laboratory of Computational Proteomics, Institute of Cytology and Genetics Siberian Branch of the Russian Academy of Sciences, Novosibirsk, Russia

**Keywords:** epitopes, conformational B-cell epitopes, protein language model, GVP, transfer learning, transformer, antibody - antigen complex

## Abstract

One of the primary tasks in vaccine design and development of immunotherapeutic drugs is to predict conformational B-cell epitopes corresponding to primary antibody binding sites within the antigen tertiary structure. To date, multiple approaches have been developed to address this issue. However, for a wide range of antigens their accuracy is limited. In this paper, we applied the transfer learning approach using pretrained deep learning models to develop a model that predicts conformational B-cell epitopes based on the primary antigen sequence and tertiary structure. A pretrained protein language model, ESM-1v, and an inverse folding model, ESM-IF1, were fine-tuned to quantitatively predict antibody-antigen interaction features and distinguish between epitope and non-epitope residues. The resulting model called SEMA demonstrated the best performance on an independent test set with ROC AUC of 0.76 compared to peer-reviewed tools. We show that SEMA can quantitatively rank the immunodominant regions within the SARS-CoV-2 RBD domain. SEMA is available at https://github.com/AIRI-Institute/SEMAi and the web-interface http://sema.airi.net.

## 1 Introduction

Selection of B-cell antibodies specifically targeting the external antigen proteins is a natural immune response *in vivo*. Corresponding antibody binding sites are called conformational B-cell epitopes, and their knowledge is important for the effective design of peptide- and protein-based vaccines and development of immunotherapeutic drugs (1). To date, multiple methods have been developed using machine learning and other approaches to predict conformational B-cell epitopes within an antigen sequence. For example, widely used tools include SEPPA3, BepiPred2.0, PEPITO, Epitopa, DiscoTope, iBCE-EL and iLBE (2–8). These tools are commonly based on conventional machine learning methods, including linear regression, random forest, support vector machines, or combinations thereof. Physico-chemical and structural properties such as atomic coordinates, relative surface accessibility, protrusion index are extracted to train the model or to derive more complex features. Existing methods demonstrate good performance, however, improving the accuracy of prediction of conformational B-cell epitopes is still of great importance. Improving the performance of conformational epitope prediction tools is a challenging task, in particular due to the limited amount of available experimental data, uncertainties in defining epitope residues and extracting reliable antigen features for model construction.

Deep learning approaches are increasingly often applied to protein analysis and design tasks. One such approach is transfer learning using latent space vector representations of amino acid residues extracted from large pretrained protein language models. These representations are able to implicitly encode context-dependent structural, functional and physico-chemical protein properties (9–11), which makes them attractive to develop new models to capture the immunogenic properties of amino acid residues.

The ESM-1v model is one of the largest transformer-based protein language models trained in a self-supervised fashion (9). Recently, the ESM-IF1 model, a sequence-to-sequence transformer with invariant geometric input processing layers, has been developed to predict the protein sequence based on its tertiary fold (12). ESM-IF1 is based on GVP-GNN (13) and generic autoregressive encoder-decoder Transformer architectures (14). The ESM-IF1 model allows to solve a variety of tasks, including inverse protein folding problem and predicting the effect of mutations.

In this paper we demonstrate that prediction of B-cell conformational epitopes can be significantly improved by applying transfer learning approaches using both pretrained ESM-1v and ESM-IF1 models. We fine-tuned ESM-1v and ESM-IF1 models to predict residues comprising B-cell epitopes by providing an interpretable score corresponding to the expected number of contacts of an amino acid residue with the target antibody.

Taking into consideration uncertainties in the epitope residue assignment we generated a benchmark that included antigens with classified epitope residues based on two distance cut-off values. The first distance defines the presence of a contact between the antigen and antibody residues, while the second distance indicates if the residue is too distant from the epitope and should be ignored in metric calculations or model training. This allows to evaluate the robustness of the model to selected cut-off radii as well as exclude ambiguous labels from the training set.

The best performing model across all benchmark tasks was called SEMA (Spatial Epitope Modelling with Artificial Intelligence). We evaluated SEMA against an independent retrospective benchmark composed of antigen residues with no prior information on antibody binding sites before the 2020 release date. SEMA was compared with BepiPred-2.0  (3), SEPPA3.0  (2), PEPITO  (4), ElliPro  (15) and DiscoTope-2.0  (6) and outperformed these tools, demonstrating the highest ROC AUC value of 0.76. The SEMA prediction score was shown to correlate with the estimated immunogenicity of epitope residues according to statistical analysis of the interaction between SARS-CoV-2 RBD domain and target antibodies. SEMA is available as an online-tool and could be used for predicting B-cell conformational epitopes.

## 2 Method

### 2.1 Benchmark generation

We generated a non-redundant conformational epitope data set based on the available data on antigen-antibody complexes in the PDB database. The pipeline used to generate the conformational epitope data set included the following steps:

(1) The ANARCI tool was used to screen sequences of protein structures published in the PDB database that comprise heavy and light chains of Fabs  (16).
(2) Heavy/light Fab pairings were identified by calculating the distances between subunit residues corresponding to heavy and light chains, and only heavy and light chains with direct contacts of non-CDR regions within the distance of 4.5 Å were considered as the heavy/light pair. CDR loops were defined using Chothia numbering based on annotation by the ANARCI tool. Identified Fab pairs were manually inspected to filter out artefacts.
(3) Protein subunits that were not annotated as an antibody and had at least 5 residues interacting with antibody residues within the radius of 4.5 Å with L1/L2/L3 or H1/H2/H3 CDR loops of antibodies were considered as antigens.
(4) The contact number was calculated as the number of interactions of antigen residue atoms with antibody atoms within a sphere of radius *R*. For each antigen residue, we considered two options for estimating the contact number based on the selected radius *R*: in the first case, we calculated the number of atoms of antibody residues in contact with any atom of antigen residue _*i*_ within the distance radius *R* (cn_atom). In the second case, we calculated the number of antibody residues in contact with any atom of antigen residue _*i*_ within the distance radius *R* (cn_aa).
(5) The calculated contact numbers were mapped on the complete amino acid sequence of the antigen. The complete sequence was extracted from PDBSeqRes records. All residues missing in the protein tertiary structure but present in the sequence were labelled as “unknown”. “Unknown” residues were excluded during model training.
(6) To avoid redundancy, antigen sequences were clustered according to the degree of sequence identity (> 95%) using MMseqs2 software  (17). MMseqs2 enables sensitive protein sequence searching for the analysis of massive data sets. Sequences from the same cluster were aligned using MAFFT  (18) and consensus epitope labels were assigned to the center of the cluster. For each residue in the reference sequence, the consensus contact number value was assigned as the maximum contact number observed among antigen-antibody complexes in the data set within an identity cluster.
(7) Each antigen residue was assigned one of the following three labels. The “epitope” label was assigned according to the distance radius R1 within which at least one Fab pair residue was observed. The following values of *R*1 were considered: 4.5, 6.0 and 8.0 Å. The “close” label was assigned when the antigen residue was located outside *R*1 but within the radius *R*2 (*R*2 > *R*1) of the closest antibody residue. The following values of *R*2 were considered: 12.0, 14.0 and 16.0 Å or infinite. Remaining antigen residues were labeled “distant”. Both “close” and “distant” residues were considered as non-epitope residues.
(8) To fairly compare the precision of SEMA with other tools, we generated a retrospective data set. The data set was split into training and test sets according to the structure release date. The test set included structures that were first released in the PDB database after January 1, 2020 with no available homologues with a degree of sequence identity of >70% before this date. The remaining antigens were divided into training and validation sets in a ratio of 9:1.

### 2.2 SEMA-1D and SEMA-3D models construction

SEMA-1D and SEMA-3D regression models were developed independently by adding a fully-connected linear layer on top of the ESM-1v and ESM-IF1 pretrained models, respectively. The last layers of the corresponding pretrained models provide amino acid residue representations with embedding size of 1280 and 512. The added fully-connected layer of each model takes an input from the last layer of the respective pretrained model and returns a one-dimensional vector. Each element of this vector is used to predict the log-scaled contact number value of corresponding amino acid residue position.

We used the Adam optimizer and the masked mean squared error loss defined as a mean squared error loss function ignoring masked residues labeled as “distant” and “unknown” residues class.

SEMA-1D and SEMA-3D models were fine-tuned independently. The SEMA-1D model was trained for two epochs with a starting learning rate of 1e – 5 and linear learning rate decay. The SEMA-3D model was trained for two epochs with a starting learning rate of 1e – 4 and linear learning rate decay.

The final models were obtained as an ensemble of five models fine-tuned independently from the same pretrained checkpoint.

As a limitation, the original ESM-1v model was pretrained with a maximum sequence length of 1022. Accordingly, sequences longer than 1022 residues were trimmed from C-terminus to the length of 1022.

### 2.3 Prediction using peer methods

Protein sequences of the tested antigens were submitted to the BepiPred 2.0 server (https://services.healthtech.dtu.dk/service.php?BepiPred-2.0), and the results were downloaded in csv format (50 per run). Discotope has a stand-alone implementation on Python and was run on our own server  (6). ElliPro has a stand-alone implementation on Java and was run on our own server  (15). Antigen structures were submitted to the BePro server, also known as PEPITO (http://pepito.proteomics.ics.uci.edu). The same PDB identifiers and chains were selected for submission to the SEPPA3.0 prediction server (http://lifecenter.sgst.cn/seppa/index.php) and score files were retrieved and used for metrics evaluation. All tools were run with default options.

### 2.4 Model performance metrics

Performance of the models was assessed using AUC, Matthews correlation coefficient (MCC), positive predictive value (PPV) and sensitivity metrics. To calculate MCC, PPV and sensitivity we converted prediction values to a binary values applying a threshold. Threshold was set as an optimal cut-off provided by ROC AUC analysis corresponding to the highest true positive rate together with the lowest false positive rate. Taking into account imbalance between epitope and non-epitope classes in the data sets, PPV and sensitivity metrics were calculated for each class, and then their unweighted mean was found.

## 3 Results

### 3.1 Benchmark for predicting epitope features

Crystallographic data on antigen-antibody structures are commonly used to identify conformational B-cell epitopes. In this paper, we screened the PDB database to select the antigen epitope residues interacting with the antibody. For each antigen residue, we calculated the contact number feature, which indicates the number of contacts of the antigen residue with antibody residues within the distance radius *R*1. The resulting benchmark generated using the pipeline (see Methods) contained a total of 4,739 records, with 884 antigen sequences clustered based on the degree of identity of 95%. The test set included 101 antigen sequences.

**Figure 1:**
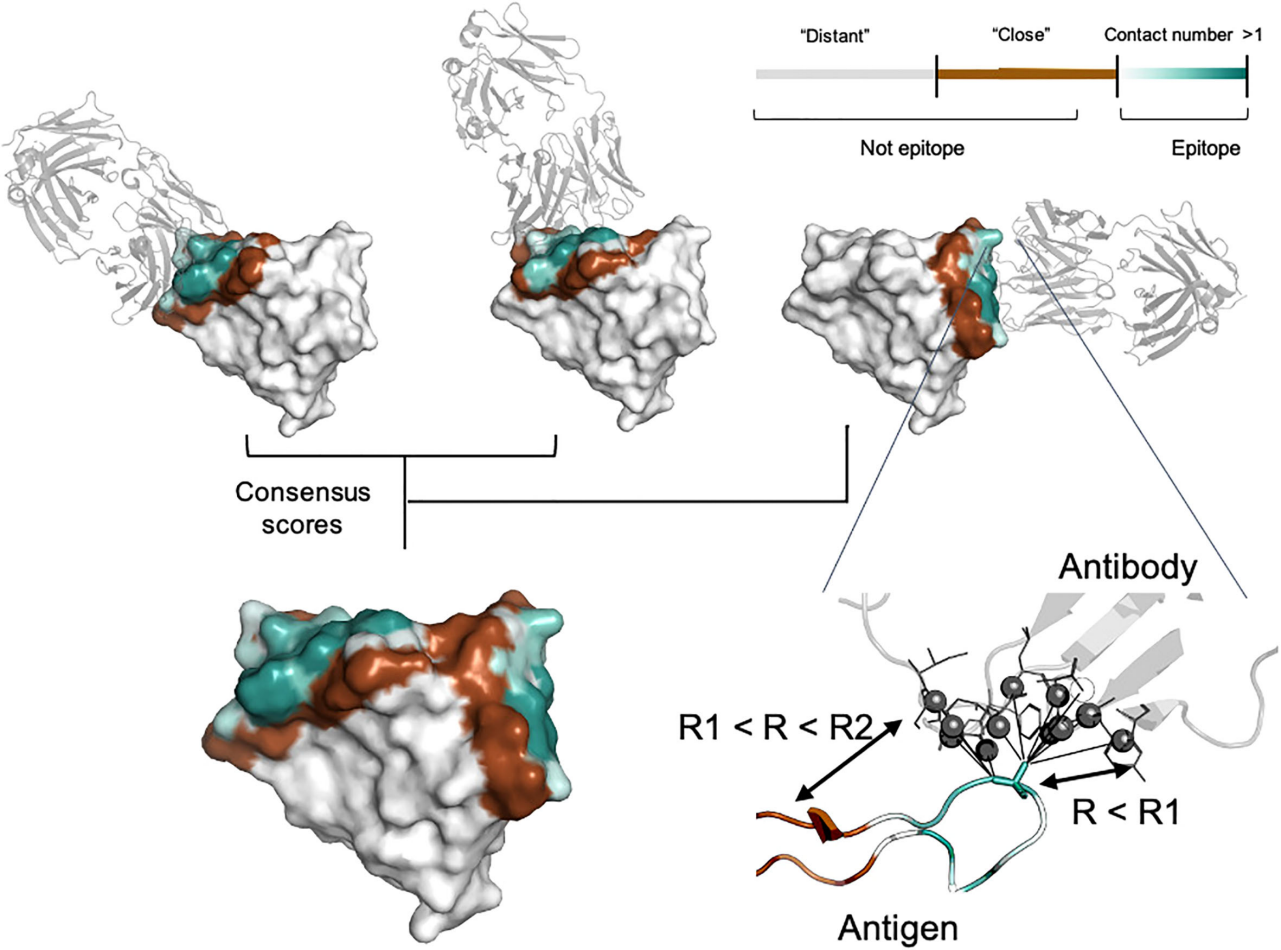
Epitope data set generation. For each residue, the contact number interaction feature was calculated, which corresponds to the log-scaled number of contacts of antigen residues with antibody residues within the radius *R*1. Antigen residues within *R*1 and *R*2 distance of the antibody were classified as non-epitope (brown color). Residues located further away than *R*2 were either considered as non-epitope (the “unmasked” data set) or ignored in the model training and and calculation of the relevant metrics (the “masked” data set, highlighted in gray). The color gradient from light cyan to dark cyan corresponds to the contact number value ranked from low to high, respectively. The color map is shown in the top right-hand corner of the figure. Epitopes obtained from distinct antigen-antibody complexes from the PDB database were merged to provide the final antigen epitopes data set.

Antigen residues were considered as epitope if the distance to the interacting antibody was lower than specified cut-off value (*R*1). *R*1 was selected in the range of 4.5, 6.0 and 8.0 Å. The cut-off value of 4.5 Å reflects the presence of direct interaction with antibody residues. Radius values of 6.0 Å and 8.0 Å additionally include residues involved in long-range interaction. It is well known that epitopes can be spatially distributed on the antigen structure and for some cases such experimental information might be missing. To take this into account, we split non-epitope residues based on the distance from the interacting antibody (*R*2) into “close” (*R* < *R*2) and “distant” (*R* > *R*2) (**Figure 1**). We selected *R*2 equal to either 12.0, 14.0 or 16.0 Å to analyze the effect of epitope boundary region information on model accuracy.

In addition to conventional classification of epitope residues, for each antigen residue we calculated the contact number interaction feature. The contact number is a measure of the number of contacts of an antigen residue with atoms of antibody residues. Contact numbers may provide an additional interpretive score reflecting how deeply the residue is buried in the antigen/antibody interface. This might improve training efficiency by providing additional spatial information to the model. Finally, we combined information on different antibodies for the same antigen into consensus mask. The summary of the generated consensus mask is shown in **Figure 1**.

This data set was used to train and evaluate the models to solve the following tasks: (1) the conventional task of binary classification of antigen residues into epitope/non-epitope residues (with both “close” and “distant” residues classed as “non-epitope”); (2) prediction of epitope residues on the “masked” data set, that includes only “epitope” residues and residues localized “close” to epitope, excluding “distant” residues from model training and metrics calculation; (3) quantitative prediction of contact number features of antigen residues. We suggest that evaluation of the model on the different data sets generated using a wide range of *R*1 and *R*2 radii could allow to evaluate the robustness of the model in terms of predicting epitope residues independent of the ambiguities in the epitope definition.

### 3.2 Fine-tuning the models and internal validation of SEMA

The generated conformational epitope data set was used to train SEMA-1D and SEMA-3D models based on pretrained ESM-1v and ESM-IF1 models to predict the contact number antigen-antibody interaction features together with binary classification into epitope/non-epitope residues. ROC AUC metric was used to estimate model performance. We analyzed the performance of the models for two groups of test sets: (1) “unmasked” test sets, in which all antigen residues were classified as epitope or non-epitope according to selected radii *R*1 (4.5, 6.0 and 8.0 Å); (2) “masked” test sets, in which all antigen residues located further than *R*2 (12.0, 14.0, 16.0 and infinity Å) from the antibody were “masked” and ignored in ROC AUC calculations.

We also evaluated sets of *R*1 and *R*2 radii values for training set generation to select a model that performs best on both “masked” and “unmasked” test sets regardless of the selected radii. Additionally, we evaluated two approaches to calculating the contact number: in the first case, we counted the number of antibody atoms contacting antigen within the radius *R*1 (cn_atom), whereas in the second case, we calculated the number of residues with at least one contact within the radius *R*1, which resulted in a lower value (cn_aa).

**Figure 2:**
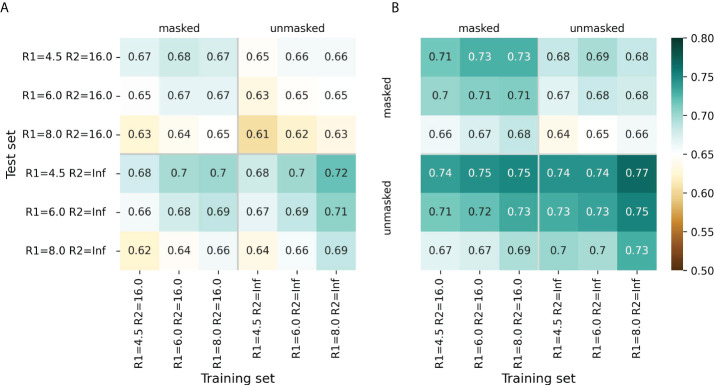
Model performance metrics estimated for the “masked” and “unmasked” test and training sets. **(A)** ROC AUC values for SEMA-1D. **(B)** ROC AUC values for SEMA-3D. The color gradient indicates the ROC AUC value from low (brown) to high (cyan).

For most radii variants, the models showed better performance when trained to predict cn_atom rather than cn_aa; this was further used for the selection of the final models (**Supplementary Figures S1**, **S2**). For both ESM-IF1 and ESM-1v, the highest ROC AUC values (0.77 and 0.72, respectively) were obtained for “unmasked” test sets with *R*1=4.5 Å (**Figure 2**, **Supplementary Figures S1**, **S2**). However, the same models exhibited worse performance for “masked” test set. This might be due to the fact that classification of antigen residues located close to epitope is a more challenging task.

The models trained on *R*1 = 8.0 Å and *R*2 = 16.0 Å achieved a robust performance on all test sets regardless of the selected radii. Finally, ROC AUC values obtained by the fine-tuned ESM-1v model was 0.7 and 0.67 for “masked” and “unmasked” test sets, respectively (**Figure 2A**). ROC AUC values for ESM-IF1 fine-tuned models were 0.75 and 0.73 for “masked” and “unmasked” test set, correspondingly (**Figure 2B**).

**Figure 3:**
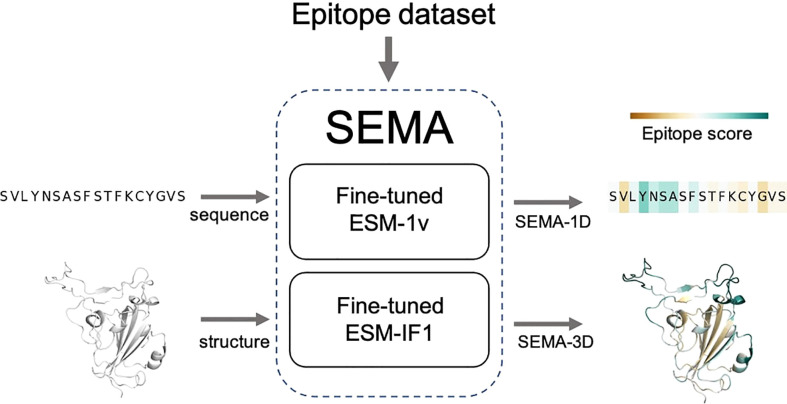
Scheme of SEMA. SEMA comprises fine-tuned ESM-1v (SEMA-1D) for the sequence-based and fine-tuned ESM-IF1 (SEMA-3D) for the structure-based prediction of epitopes.

The final fine-tuned models were called SEMA. SEMA involves the use of sequence-based (SEMA-1D) and structure-based (SEMA-3D) approaches to predict the conformational B-cell epitopes and provide an interpretable score indicating the log-scaled expected number of contacts with antibody residues (**Figure 3**).

**Figure 4:**
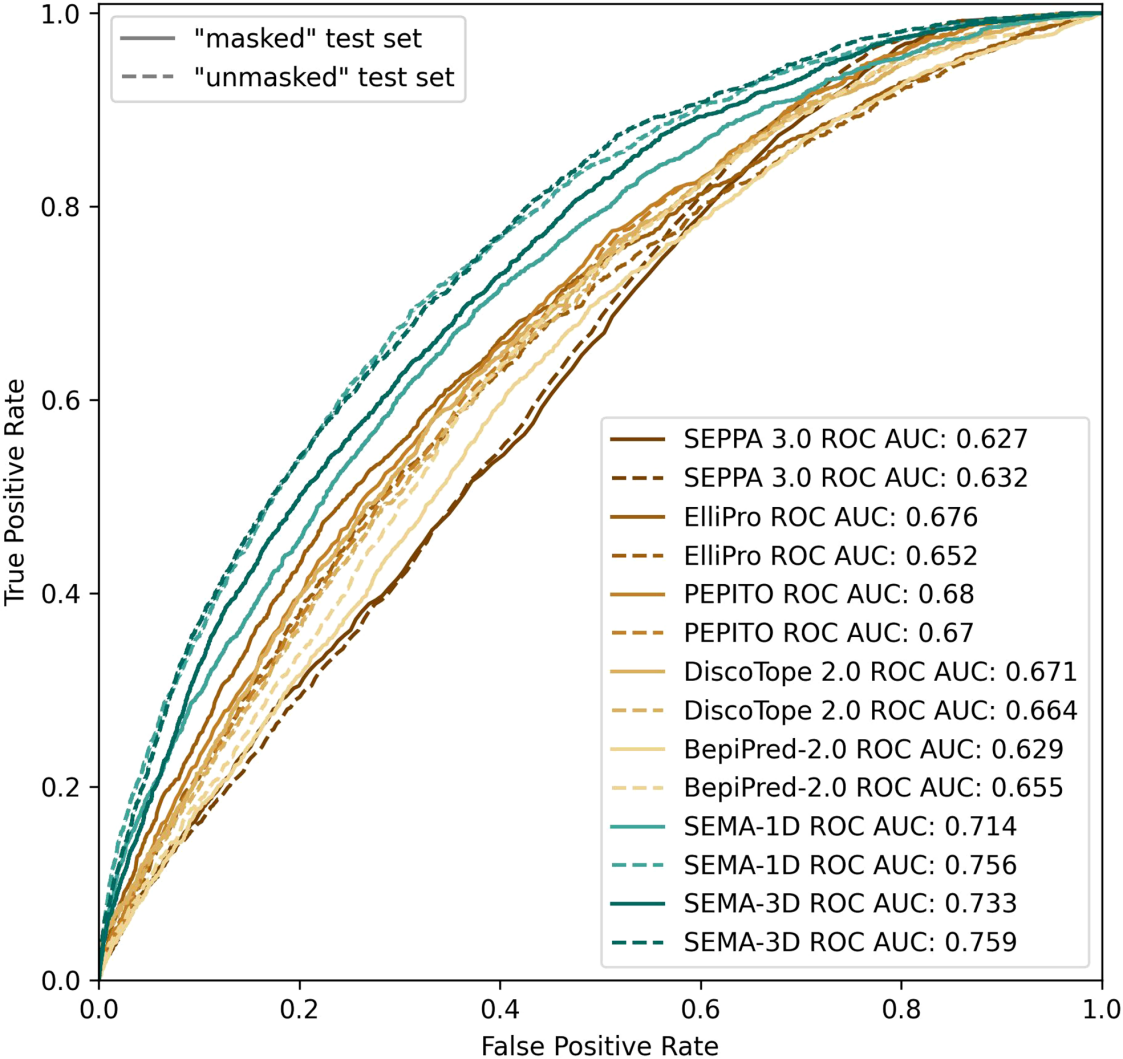
ROC AUC metrics calculated using peer methods, SEMA-1D and SEMA-3D on two test sets: “masked” (*R*1 = 4.5 Å; *R*2 = 16.0 Å) and “unmasked” (*R*1 = 4.5Å; *R*2 = *infinite*).

We found that calculating an ensemble of models obtained with different initialization parameters led to a noticeable improvement in the prediction of epitope residues. Thus, the final model was obtained as an ensemble of five models averaging their results. SEMA-1D achieved the best ROC AUC of 0.76/0.71 on “unmasked” and “masked” test sets respectively, whereas the SEMA-3D achieved best ROC AUC score of 0.76/0.73 (**Figure 4**).

### 3.3 Comparison with peer methods

The list of peer methods included BepiPred-2.0  (3), DiscoTope 2.0  (6), PEPITO  (4), Epitopia (5) and SEPPA 3.0  (2). Model performance was evaluated against collected benchmarks (see Methods). Benchmarks included antigens with no prior information in the PDB database before January 1, 2020. These cases were not included in any training set, which enabled a fair comparison of tools with each other.

We compared the performance metrics including AUC, MCC, PPV and sensitivity for both “masked” and “unmasked” test sets (**Table 1**, **Figure 4**). For the test set classification into epitope and non-epitope residues, *R*1 was set equal to 4.5 Å, which was also used in the training set for other tools. In the masked test set, *R*2 was set to 16.0 Å.

**Table 1:** Performance comparison of SEMA-1D and SEMA-3D with other peer methods on two test sets: “masked” ( *R*1 = 4.5 Å; *R*2 = 16.0 Å) and “unmasked” (*R*1 = 4.5 Å; *R*2 = *Infinite*).

Test set	Method	AUC	Threshold	MCC	PPV	Sensetivity
	SEPPA 3.0	0.627	0.048	0.176	0.772	0.436
	ElliPro	0.676	0.582	0.218	0.754	0.645
	PEPITO	0.680	0.110	0.218	0.756	0.559
“masked”	DiscoTope 2.0	0.671	-10.954	0.206	0.748	0.626
	BepiPred-2.0	0.629	0.485	0.167	0.747	0.548
	SEMA-1D	0.714	0.491	0.258	0.774	0.639
	SEMA-3D	0.733	1.210	0.269	0.778	0.646
	SEPPA 3.0	0.632	0.048	0.133	0.890	0.392
	ElliPro	0.652	0.582	0.128	0.883	0.621
	PEPITO	0.670	0.070	0.144	0.884	0.506
“unmasked”	DiscoTope 2.0	0.664	-13.130	0.132	0.890	0.500
	BepiPred-2.0	0.655	0.485	0.133	0.888	0.560
	SEMA-1D	0.756	0.491	0.217	0.900	0.689
	SEMA-3D	0.759	1.029	0.202	0.905	0.593

Threshold is an optimal cut-off chosen by ROC AUC analysis corresponding to the highest true positive rate together with the lowest false positive rate. AUC – area under curve, MCC – Matthews correlation coefficient, PPV – positive predictive value.

The results show that the sequence-based methods, as well as SEPPA 3.0 and SEMA-3D perform better on conventional epitope prediction task on the “unmasked” test set compared to the “masked” test set. This indicates poorer performance in classification of non-epitope residues located close to epitope and predicting epitope borders. In contrast, PEPITO, ElliPro and Discotope 2.0 tools demonstrate the highest ROC AUC value on the “masked” test set (**Figure 4**). Compared to other methods, both SEMA-1D and SEMA-3D models have the highest performance metrics across all the benchmark tasks. The results of predictions for other values of *R*1 and *R*2 exhibit a similar trend and are shown in Supplementary table (see **Supplementary Tables S1**, **S2**). As expected, in line with SEMA, all tools have lower AUC, PPV and sensitivity values and higher MCC values for higher *R*1 radii values, while selecting finite *R*2 (*R*2 < *Inf*) radius does not significantly affect the results.

### 3.4 Case study: Prediction of immunodominant regions of the SARS-CoV-2 RBD domain

The RBD domain of the S-protein of SARS-CoV-2 is one of the most well structurally characterized antigens to date. We conducted an analysis of the RBD domain instead of the full-length S-protein to exclude the putative effects of glycosylation that is currently not considered in the SEMA  (19). To evaluate the performance of SEMA, during model training we excluded all sequences homologous to S-protein (with a degree of identity of > 70%), in particular S-proteins of MERS and SARS-CoV. SEMA-3D was evaluated on the three following tasks: (1) correctly assigning epitope and non-epitope residues; (2) correctly predicting the contact number features; (3) predicting the immunodominant epitope residues. The immunodominant residues of the RBD were estimated according to the ratio of RBD/antibody complexes in the PDB database, in which the RBD residue was in direct contact with antibody. We assume that the calculated ratio allow to estimate the immunogenicity of RBD residues, with a high ratio corresponding to immunodominant residues.

**Figure 5:**
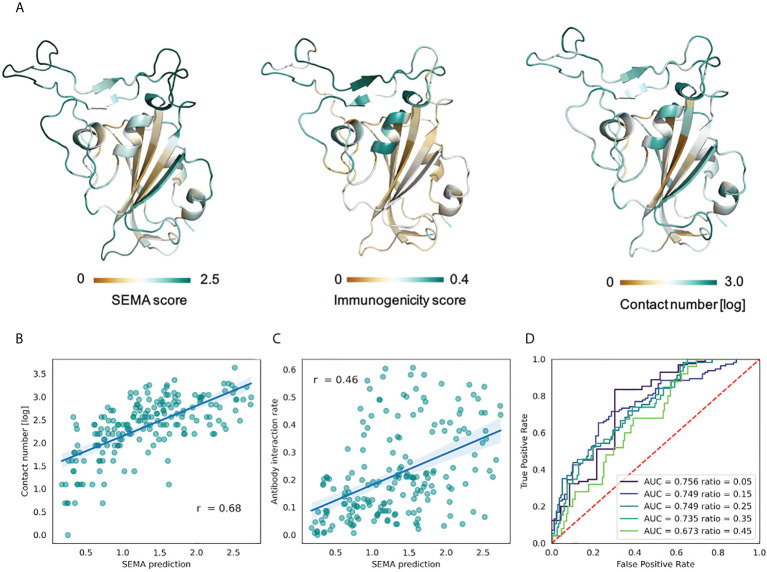
Prediction of RBD immunodominant epitopes with SEMA. **(A)** RBD domain of SARS-CoV-2 (PDB ID: 7KS9, chain B) colored according to the SEMA predicted score (left), immunogenicity score (center) and contact number values (right). Residues colored from brown (low value) to cyan (high value). Immunogenicity was estimated as the ratio of RBD/antibody complexes in the PDB database in which RBD residue was in contact with antibody within 8.0 (Å) **(B)** Correlation between the SEMA score and the log-scaled antigen contact number feature. Pearson correlation coefficient is shown. **(C)** Correlation between the SEMA score and the immunogenicity score. Pearson correlation coefficient is shown. **(D)** ROC AUC values calculated for different epitope/non-epitope residue classification based on the immunogenicity score threshold. ROC AUC values and threshold values for classification are denoted.

As shown in the **Figure 5**, SEMA-3D provides high correlation coefficients for both contact number values and estimated immunogenicity score. Additionally, we calculated the ROC AUC metrics of the model to differentiate immunodominant residues (high ratio) from other residues (low ratio), based on the ratio threshold. This provides a more reliable estimation of model performance, since most of the solvent-exposed residues of the RBD domain are labeled as epitope due to the presence of at least one structure where corresponding residues interact with the antibody. As can be seen from the score cut-off values, SEMA-3D achieves the average ROC AUC score of 0.75 on this task.

### 3.5 Web-interface

**Figure 6:**
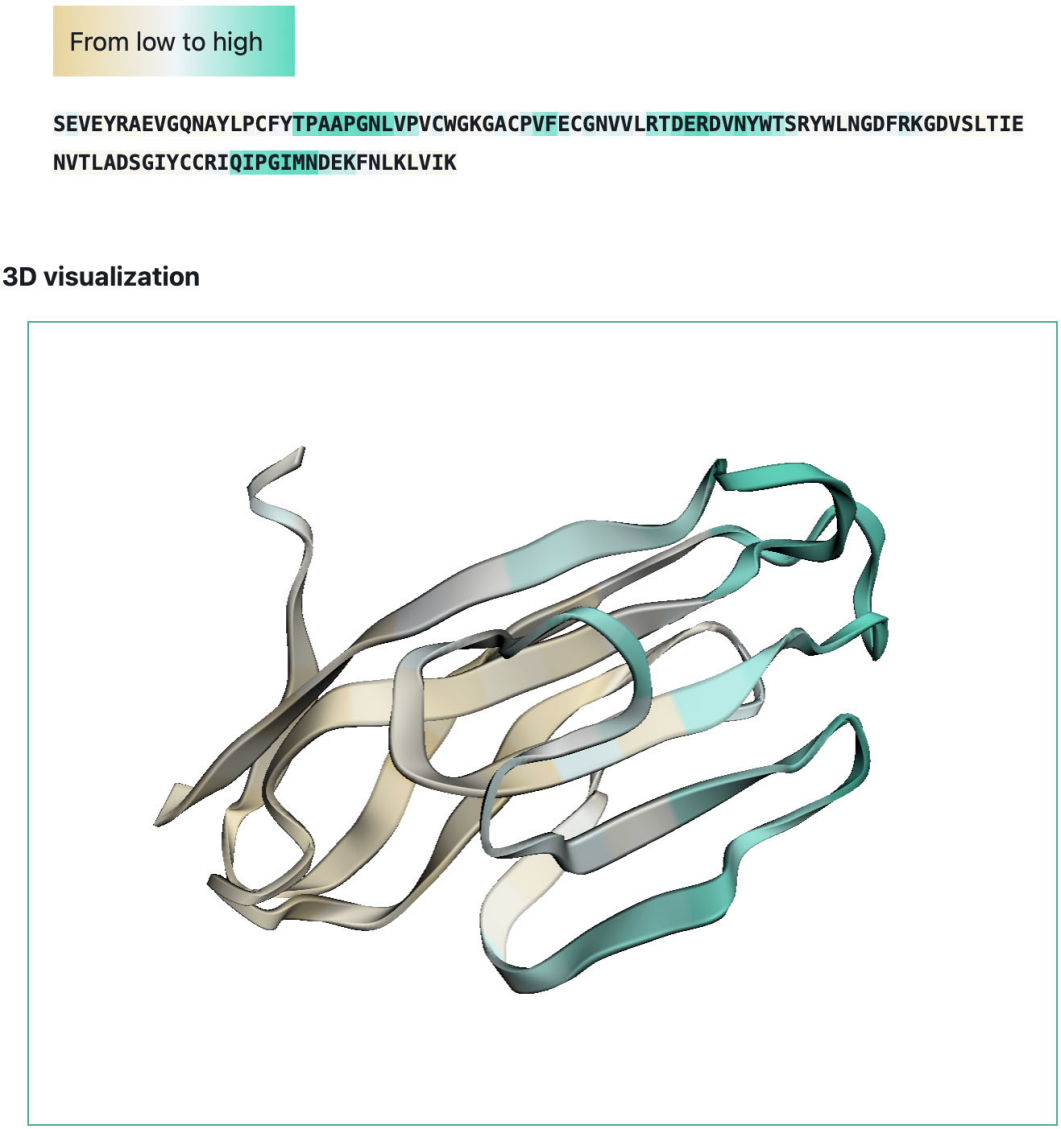
Example of SEMA-3D graphical output (PDB ID: 6TXZ, chain D). Residues are colored from brown (non-epitope) to cyan (epitope).

We developed a web-interface (http://sema.airi.net) for convenient usage of SEMA. A user can either submit a protein sequence to run SEMA-1D (fine-tuned ESM-1v model) or a protein structure in the PDB format to run SEMA-3D (fine-tuned ESM-IF1 model). The output includes predicted epitope scores for each residue in the protein sequence. To visualize the results, the output sequence in the web-interface is colored based on the predicted contact number, with colors ranging from brown (non-epitope) to cyan (epitope) (**Figure 6**). In case of SEMA-3D, output includes 3D structure of protein colored using the same color scheme as in SEMA-1D (**Figure 6**). A user can download the results in JSON and CSV format. We also provide the code of the model implemented as the Jupyter Notebook on GitHub available *via* link https://github.com/AIRI-Institute/SEMAi. We encourage using this implementation for comprehensive analysis including multiple protein sequences.

## 4 Discussion

Computational prediction of conformational epitopes is of great importance for vaccine design and therapeutics development. Conformational epitope residues are conventionally defined by a distance cut-off radius between antigen and antibody residues in the interacting complex. A residue is classified as non-epitope if it does not interact with the antigen. However, an arbitrary choice of a distance cut-off radius might lead to ambiguity in labeling epitope residues. Additionally in case of limited availability of data on the analyzed antigen, negative labels might be assigned incorrectly. In particular, for the S-protein of SARS-CoV-2, crystallographic analysis initially discovered epitopes on the RBD domain (20), but later immunodominant epitopes within the NTD domain and other regions of the S-protein were also identified (21).

To take this problems into account, we generated a benchmark that included antigens with classified epitope residues based on two distance cut-off values. The first distance, *R*1, defined the positive epitope label class, while the second distance, *R*2, defined if the residue was too remote from the epitope and was ignored in metric calculations. Finite *R*2 radii made it possible to evaluate the model’s ability to predict the boundaries of epitopes. Additionally, for each antigen residue we calculated the contact number feature corresponding to the number of atoms of the antibody located within the radius *R*1 of antigen residue. This feature was introduced for model training to provide additional spatial information on interaction between the antibody and antigen. Moreover, this feature alone was demonstrated to be a good predictor of epitope residues for a wide range of *R*1 values.

Transfer learning has been proven to be an efficient approach in the case of a limited set of examples  (22). In this paper, we show that a fine-tuned protein language model (ESM-1v) and an inverse folding model (ESM-IF1) perform well when predicting conformational epitopes. More specifically, the model was fine-tuned on a non-redundant set of only 783 antigen records with epitope residues assigned according to available antigen/antibody structures in the PDB database and selected *R*1 and *R*2 radii values.

To fine-tune the model, we screened various training sets generated using a wide range of *R*1 and *R*2 radii values and selected the model that performed best across all benchmark tasks. The final model was called SEMA; it comprises SEMA-1D (fine-tuned ESM-1v) and SEMA-3D (fine-tuned ESM-IF1) models for sequence-based and structure-based conformational B-cell epitopes prediction, respectively. SEMA was trained on the masked data set with *R*1 = 8.0 Å and *R*2 = 16.0 Å.

The high performance of SEMA can be explained by ability of protein language models to capture multiple structural and functional protein properties necessary to predict the epitope residues. In this work SEMA model was trained to automatically derive the immunogenic properties of antigen residues from representations of fine-tuned protein language models. In particular, we show that SEMA can be applied to predict immunogenicity of the RBD domain residues.

In this study, we developed B-cell conformational epitope prediction tools SEMA-1D and SEMA-3D based on the pretrained ESM-1v protein language model and ESM-IF1 inverse folding model. SEMA-1D/3D provides an interpretable score correlated with immunogenic properties of the antigen. This model demonstrated robust performance and can be applied for a wide range of antigen analysis tasks.

## Data availability statement

The original contributions presented in the study are included in the article/**Supplementary Material**. Further inquiries can be directed to the corresponding author.

## Author contributions

TS and NI carried out model training, data set generation and analysis. DU wrote the code for models fine-tuning. TS and AK carried out the analysis of published epitope tools performance. IL carried out the data set analysis. DS, MA, PS, TS and NI carried out manuscript writing. MS and TS developed web-interface. OK, DS, MA, NI designed the study. All authors contributed to the article and approved the submitted version.

## Conflict of interest

Author’s DU and IL were employed by Sber.

The remaining authors declare that the research was conducted in the absence of any commercial or financial relationships that could be construed as a potential conflict of interest.

## Publisher’s note

All claims expressed in this article are solely those of the authors and do not necessarily represent those of their affiliated organizations, or those of the publisher, the editors and the reviewers. Any product that may be evaluated in this article, or claim that may be made by its manufacturer, is not guaranteed or endorsed by the publisher.
