# Supplementary material for: SEMA: Antigen B-cell conformational epitope prediction using deep transfer learning
Source: Front Immunol. 2022 Sep 15;13:960985. doi: 10.3389/fimmu.2022.960985 (PMC9523212; doi:10.3389/fimmu.2022.960985)
Supplement: Supplementary file 1 [file DataSheet_1.pdf]

Supplementary Material

1 SUPPLEMENTARY FIGURES

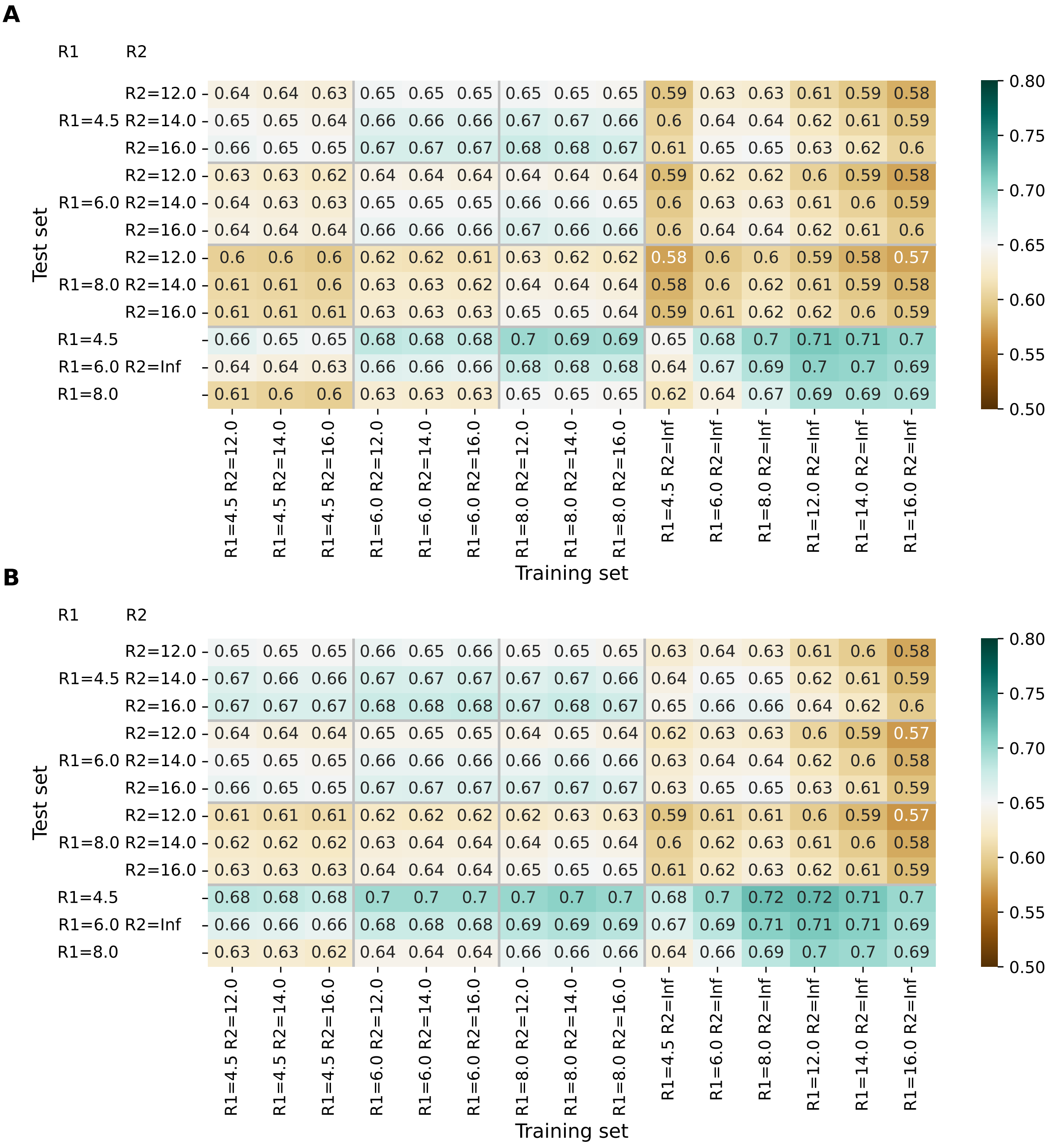

Figure S1. ROC AUC values for SEMA-1D regression model for different data set. (**A**) ROC AUC values for SEMA-1D regression model based on cn_aa approach. (**B**) ROC AUC values for SEMA-1D regression model based on cn_atom approach.

A

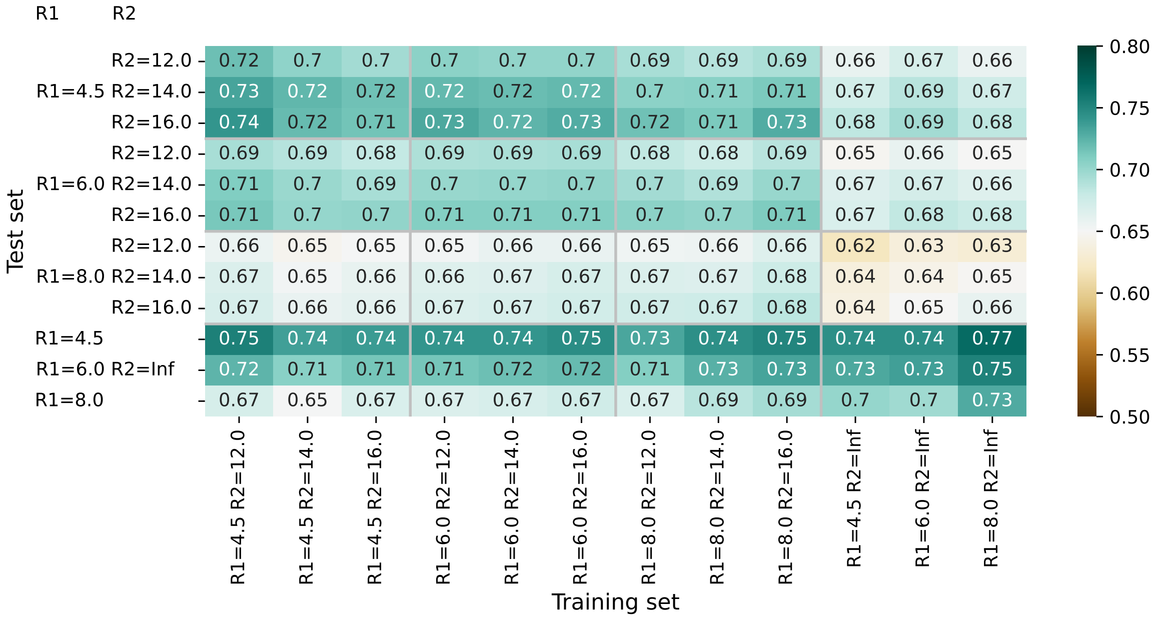

B

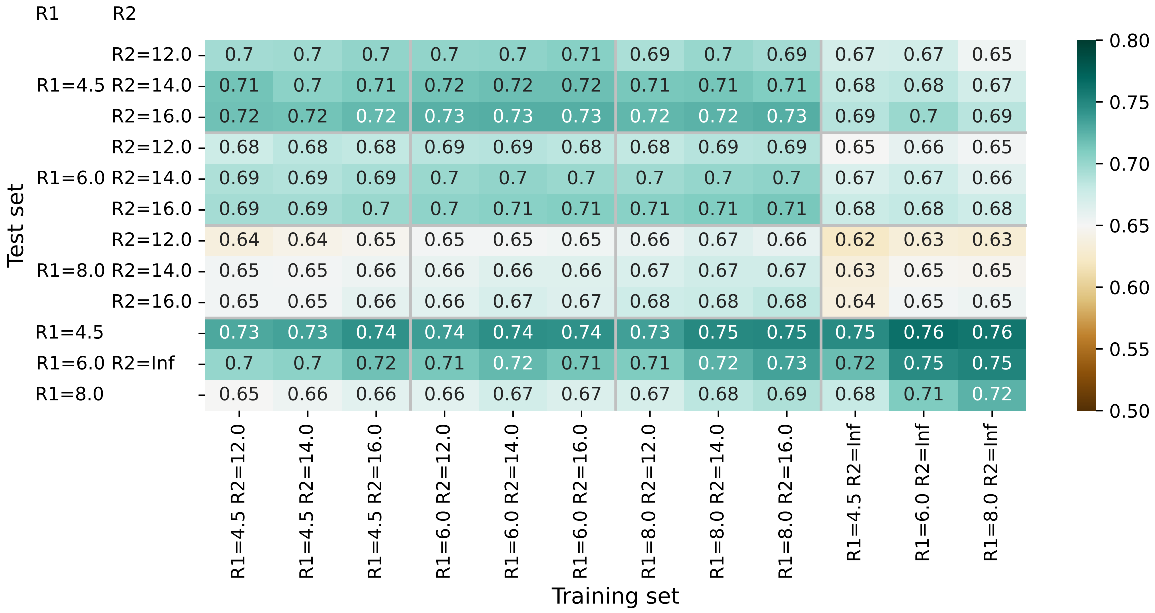

Figure S2. ROC AUC values for SEMA-3D regression model for different data set. (**A**) ROC AUC values for SEMA-3D regression model based on cn_aa approach. (**B**) ROC AUC values for SEMA-3D regression model based on cn_atom approach.
